# Supplementary material for: In vitro protease cleavage and computer simulations reveal the HIV-1 capsid maturation pathway
Source: Nat Commun. 2016 Dec 13;7:13689. doi: 10.1038/ncomms13689 (PMC5159922; doi:10.1038/ncomms13689)
Supplement: Supplementary Information — Supplementary Figures and Supplementary Table [file ncomms13689-s1.pdf]

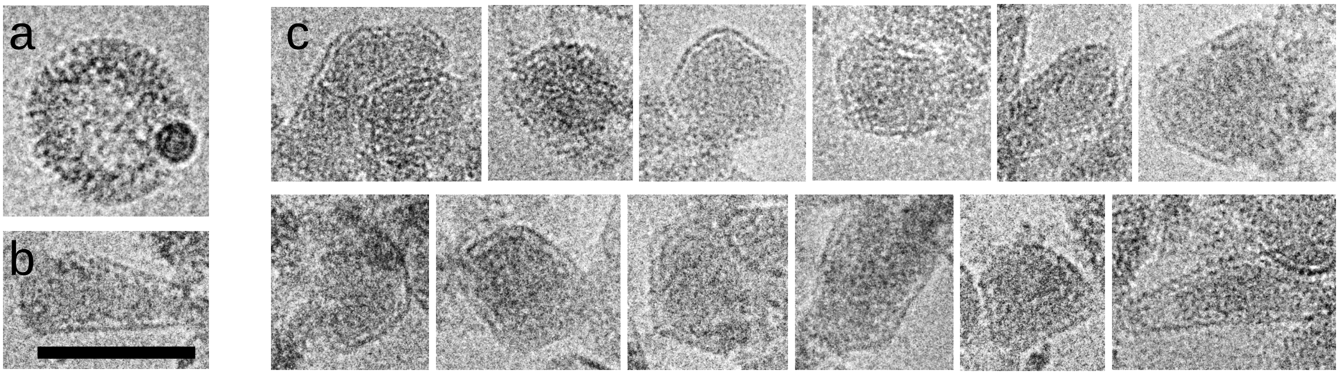

Supplementary Figure 1. Maturation intermediates by *in vitro* protease cleavage of Gag VLPs. (a) A typical Gag VLP before PR cleavage. (b) Formation of the mature core after PR treatment. (c) A gallery of “hybrid” entities resulting from incomplete maturation process. Shown are the changes from spherical immature surfaces to sharply curved surfaces. The images were recorded from the same sample in Fig. 1c labeled “*2”. All panels are on the same scale. Scale bar, 100 nm.

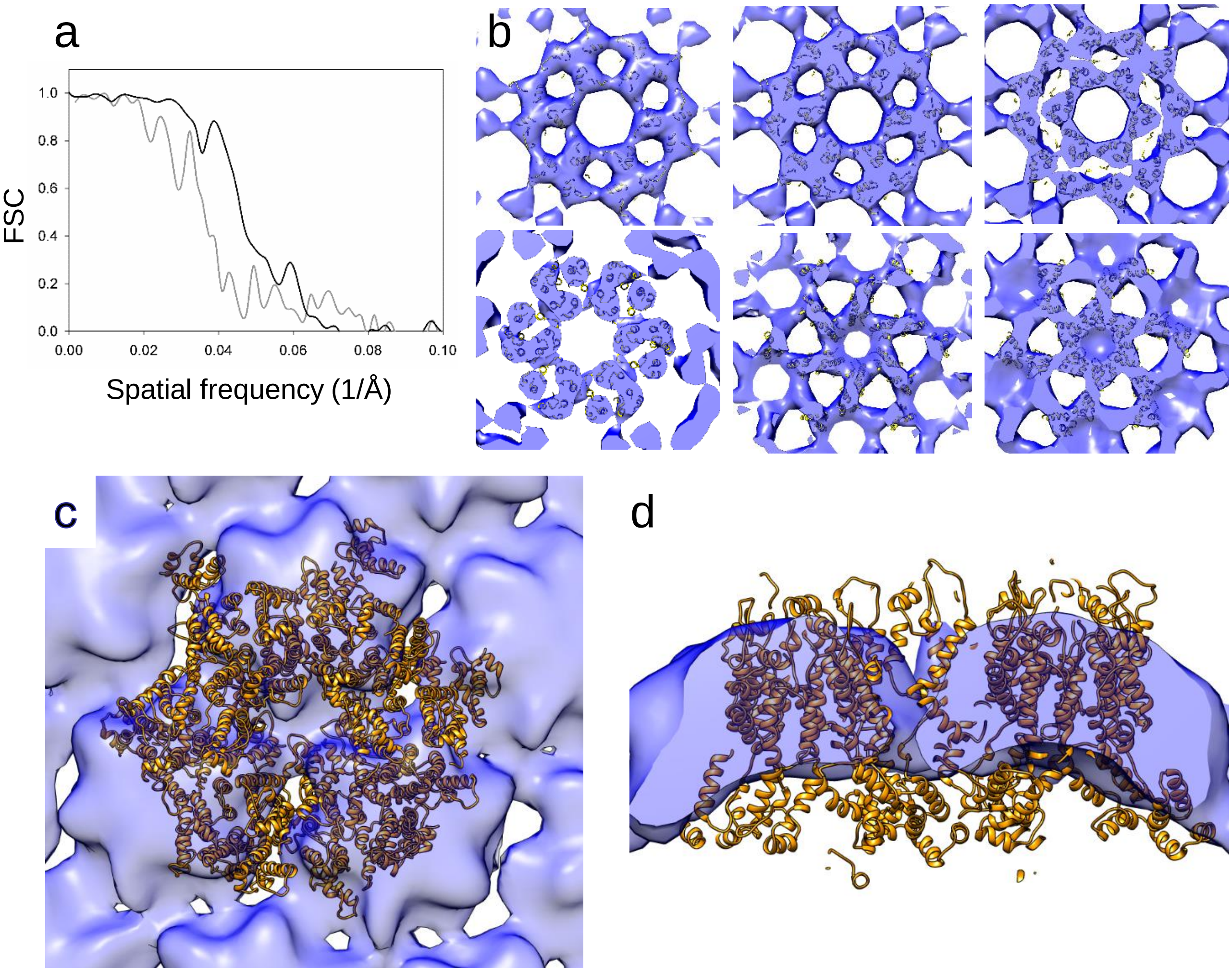

Supplementary Figure 2. (a) Fourier Shell Correlation (FSC) of the 3D density maps of Gag spheres (black) and post-cleavage tubular products (grey). (b) Fitting of immature CA lattice (PDB code 4USN) into the Gag sphere density map, shown in slice-views along the radial direction. (C&D) Fitting of immature CA lattice (PDB code 4USN) into the tube density map, viewed from tube surface (c) and along the tube axis (d). The maps are contoured at 1.5 σ.

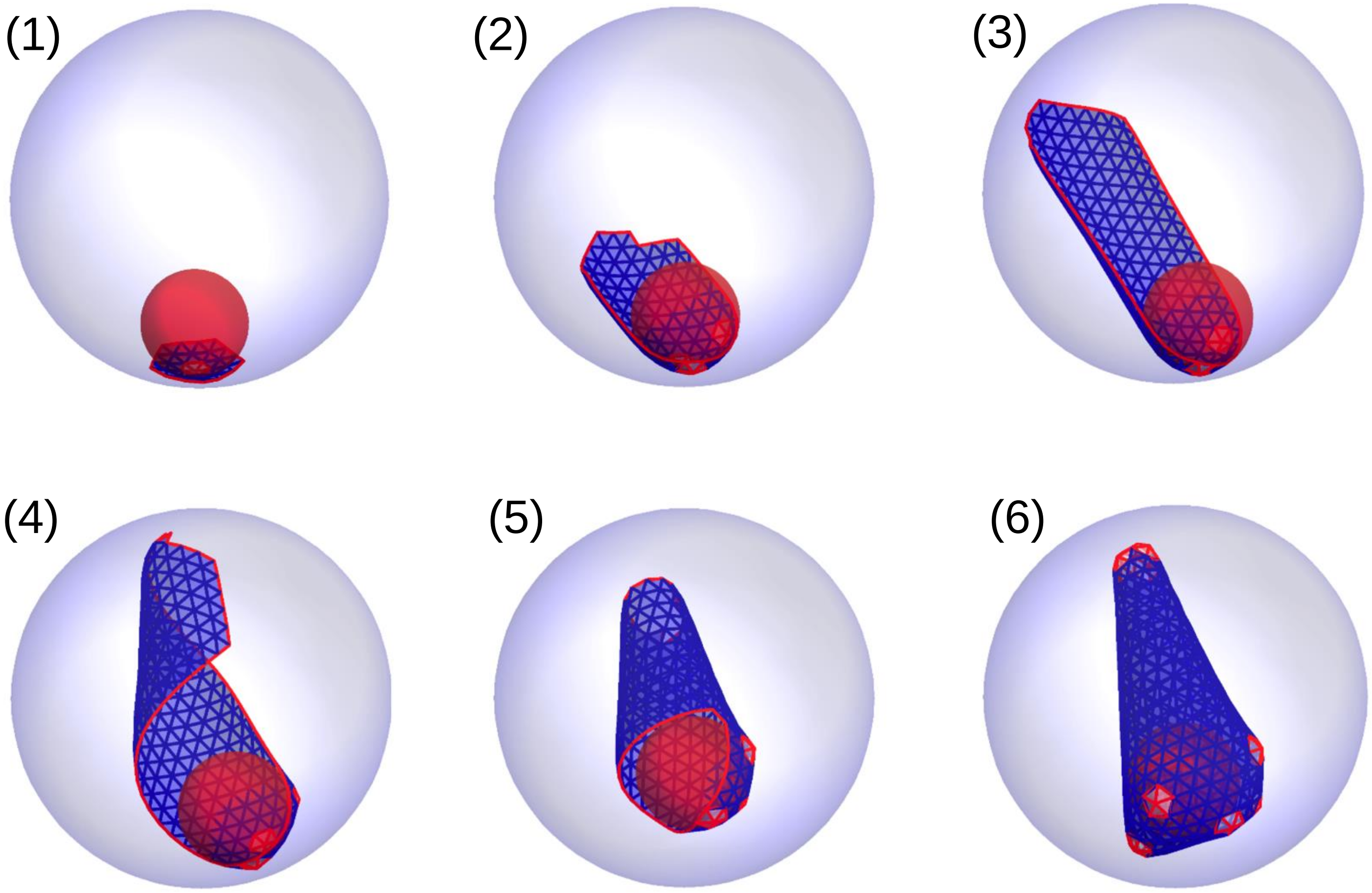

Supplementary Figure 3. Formation of a conical capsid. The genome and a few subunits remain attached to the membrane at the beginning of the growth process.

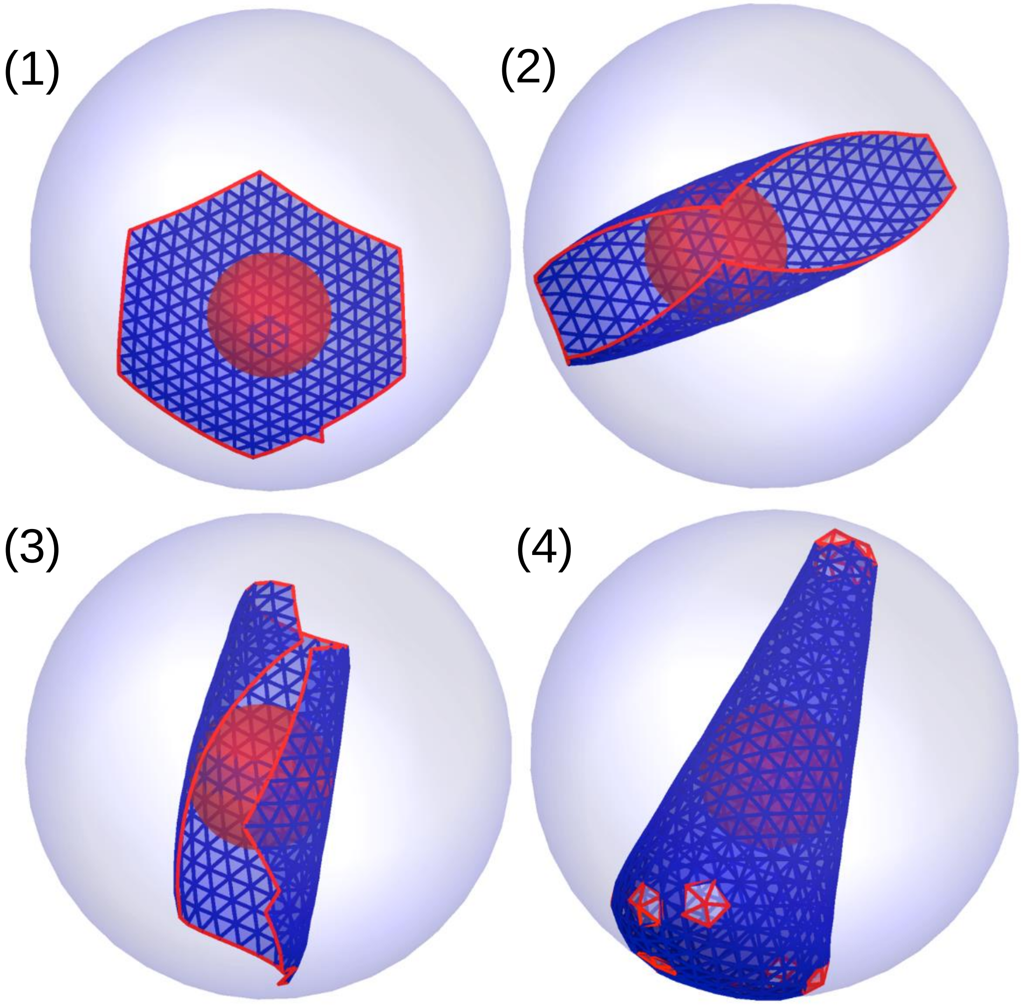

Supplementary Figure 4. Snapshots of formation of a conical capsid through a combination of displacive and disassembly/reassembly models. Initially a lattice formed from 400 hundred subunits detaches from the membrane. The lattice undergoes maturation, i.e., the mechanical properties and the spontaneous curvature of lattice transforms from an immature capsid to a mature one. The orange ball illustrates the genome.

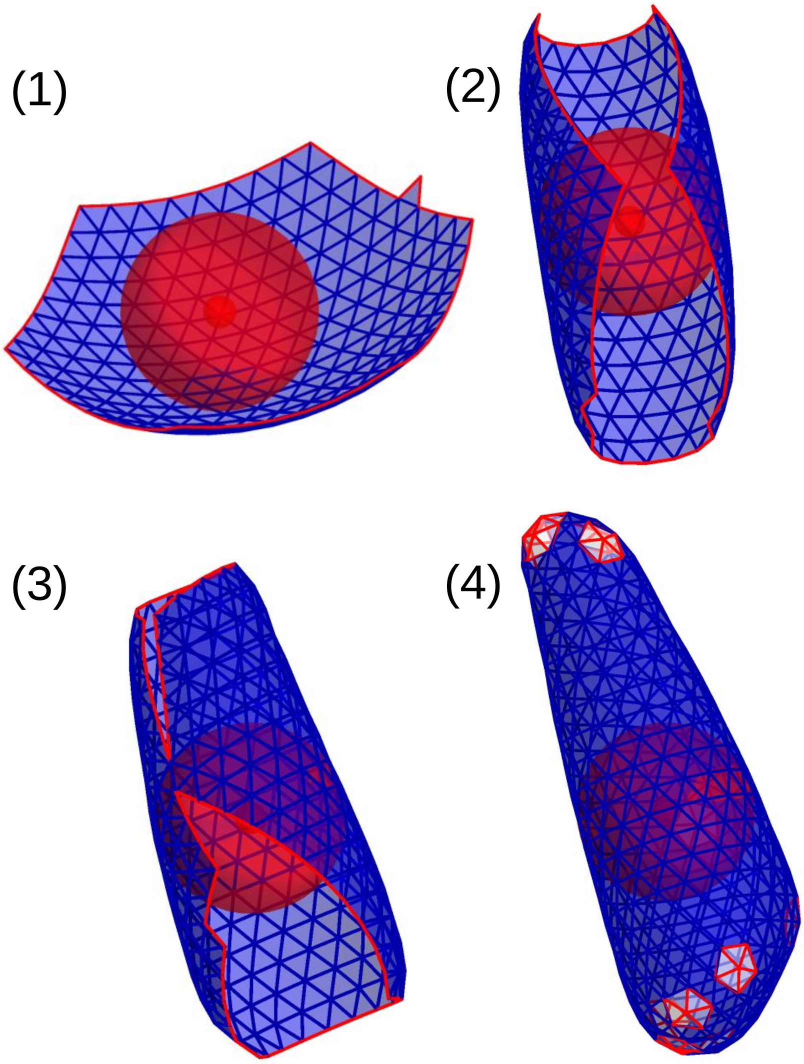

Supplementary Figure 5. Snapshots of formation of a conical capsid through a combination of displacive and disassembly/reassembly models in the absence of membrane but the presence of genome.

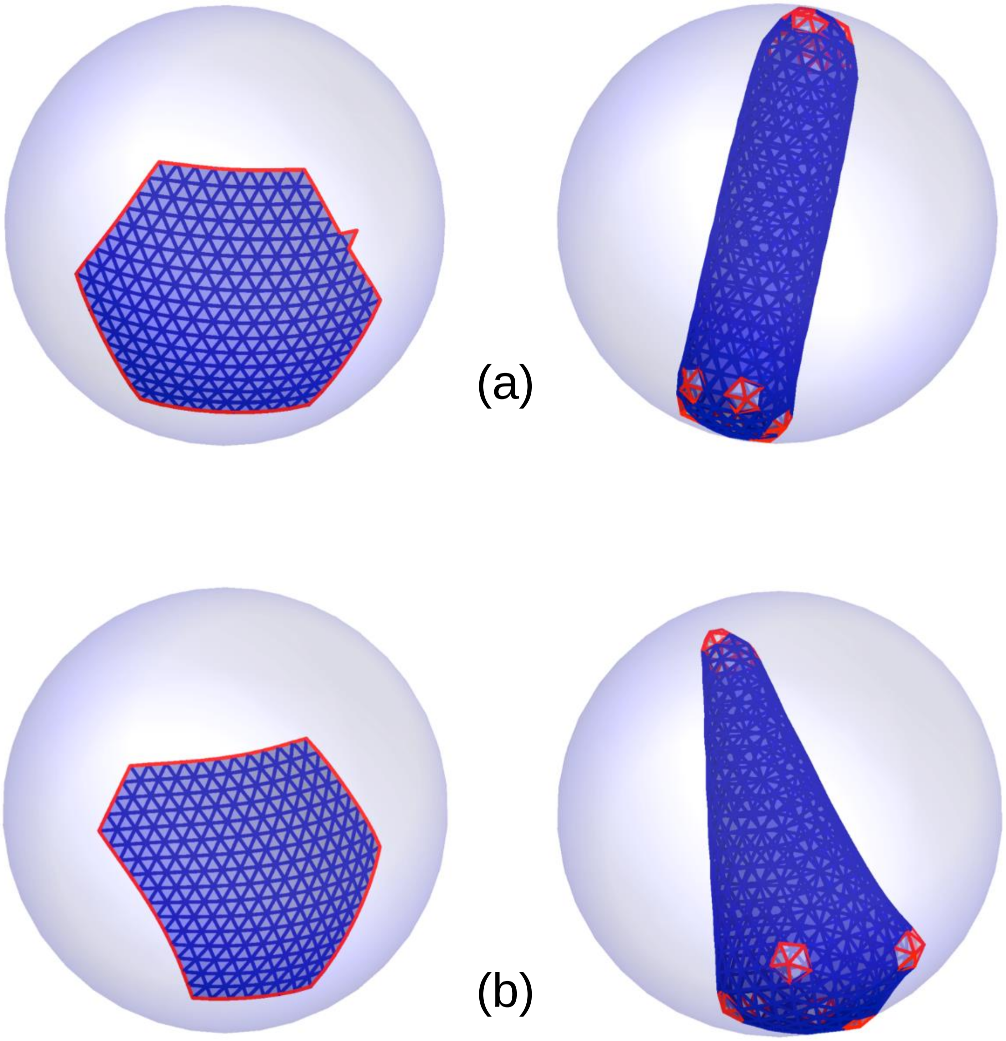

Supplementary Figure 6. The role of initial condition and symmetry in the final structure of capsids. If the initial lattice has hexagonal symmetry, a cylindrical shell forms (a); otherwise a conical capsid forms (b).

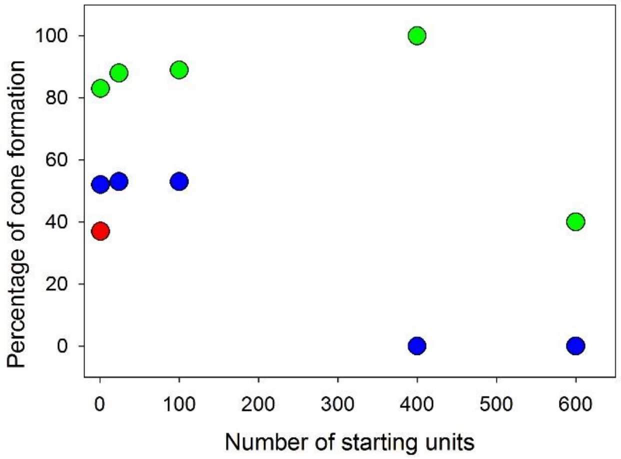

Supplementary Figure 7. Relative frequency of cone formation in simulations under the indicated conditions: in the absence of membrane and genome (red); in the presence of membrane but absence of genome (blue); and in the presence of both membrane and genome (green).

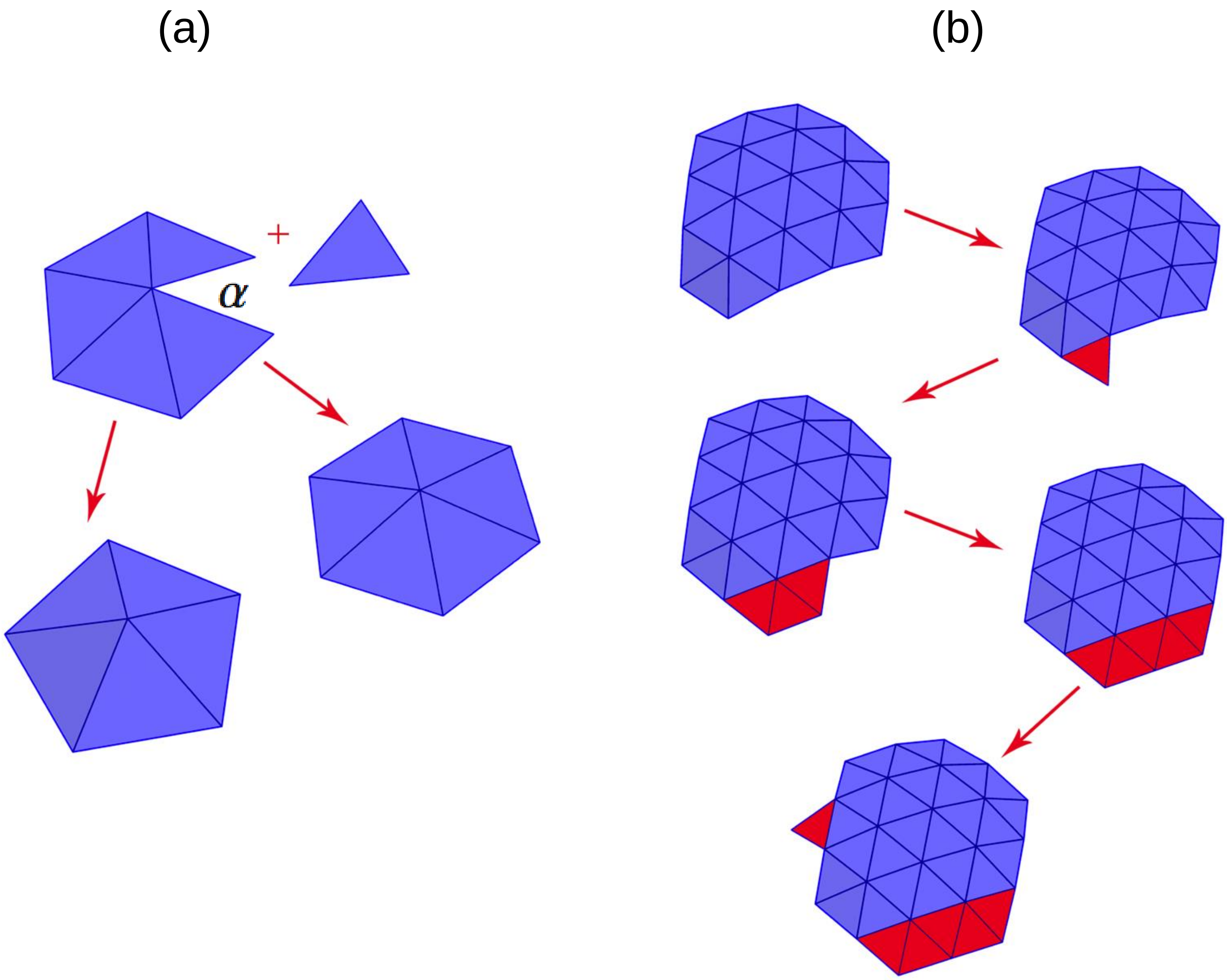

Supplementary Figure 8. (a) Formation of a pentamer or hexamer depends on the opening angle. Five assembled subunits can either become a pentamer or a new subunit can be added to them to become a hexamer. (b) The step by step growth of a shell by addition of triangular subunits.

Supplementary Table 1: Percentage of cones in total number of capsids (cones+cylinders).

**Number of CA subunits that initially transform from an immature lattice to the mature lattice without disassembly**	**Absence of membrane and genome**	**Presence of membrane but absence of genome**	**Presence of both membrane and genome**
**disassembly/reassembly**			
1 subunit	37%	52%	83%
**displacive + reassembly**			
24 subunits		53%	88%
100 subunits		53%	89%
400 subunits		0%	100%
600 subunits		0%*	40%**

*Rolling sheet.
**Some cones are defective.
